# The Effect of Nitrogen Input on Chemical Profile and Bioactive Properties of Green- and Red-Colored Basil Cultivars

**DOI:** 10.3390/antiox9111036

**Published:** 2020-10-23

**Authors:** Luís R. O. Cruz, Ângela Fernandes, Francesco Di Gioia, Spyridon A. Petropoulos, Nikolaos Polyzos, Maria Inês Dias, José Pinela, Marina Kostić, Marina D. Soković, Isabel C. F. R. Ferreira, Lillian Barros

**Affiliations:** 1 Centro de Investigação de Montanha (CIMO), Instituto Politécnico de Bragança, Campus de Santa Apolónia, 5300-253 Bragança, Portugal; luis.cruz@unipiaget-angola.org (L.R.O.C.); afeitor@ipb.pt (Â.F.); maria.ines@ipb.pt (M.I.D.); jpinela@ipb.pt (J.P.); iferreira@ipb.pt (I.C.F.R.F.); 2 Departamento de Ciências da Saúde, Instituto Superior Politécnico Jean Piaget de Benguela, Estrada Nacional 100 Lobito, Benguela 1393, Angola; 3 Department of Plant Science, Pennsylvania State University, 207 Tyson Building, University Park, PA 16802, USA; fxd92@psu.edu; 4 Department of Agriculture Crop Production and Rural Environment, University of Thessaly, Fytokou Street, N. Ionia, 38446 Magnissia, Greece; npolyzos@uth.gr; 5 Institute for Biological Research “Siniša Stanković”-National Institute of Republic of Serbia, University of Belgrade, Bulevar despota Stefana 142, 11000 Belgrade, Serbia; marina.kostic@ibiss.bg.ac.rs (M.K.); mris@ibiss.bg.ac.rs (M.D.S.)

**Keywords:** antimicrobial properties, antioxidant activity, bioactive compounds, nitrogen fertilization, *Ocimum basilicum* L., phenolic compounds, tocopherols, omega-3 fatty acids

## Abstract

In the present study, three red-colored (Dark Opal, Basilico Rosso, and Red Basil) and one green-colored landrace (Mitikas) of basil (*Ocimum basilicum* L.) were grown under four nitrogen regimes, namely Control (no fertilizer added), 200 ppm, 400 ppm, and 600 ppm of nitrogen (N). Fresh yield varied depending on N input following a quadratic function in all four genotypes, and green basil performed better compared to the red cultivars. A significant interaction of genotype × N input was recorded for most of the chemical parameters measured. Tocopherols contents of leaves were consistently higher in plants that received 200 ppm of N and lower in those receiving 600 ppm of N, especially in Dark Opal and Red Basil cultivars. Polyunsaturated fatty acids (PUFA) were the major category of fatty acids and Red Basil had the lowest ratio of omega-6/omega 3 (0.29) and thus the best fatty acid profile. Polyphenols content was the highest in Red Basil and Dark Opal (25 mg/g of extract on average) and the lowest in Mitikas and decreased with increasing N input. Similarly, antioxidant activity was the highest in Dark Opal and Red Basil fertigated with 200 ppm of N, whereas all the leaf extracts tested had good antibacterial and antifungal activity. In conclusion, basil chemical and bioactive profile was significantly influenced by both genotype and N input. Red-colored basil, although less productive, had the best chemical profile, and moderate levels of N input may provide the best compromise between yield, nutritional value, and bioactivity for the species.

## 1. Introduction

*Ocimum basilicum* L. is an important herb that belongs to the Lamiaceae family, and has high commercial interest due to its wide range of industrial uses [1,2]. Conventionally, basil leaves have been used for medicinal purposes in the treatment of coughs, headaches, constipation, diarrhea, worms, warts, and kidney problems, activities which are associated with the occurrence of phenolic compounds and anthocyanins [3,4,5]. The antioxidant properties of basil leaf extracts are also attributed to polyphenol content, while basil leaves and flowers are also rich in essential oils, which is highly appreciated by the food and pharmaceutical industries.

The phytochemicals profile in basil leaves may show a great variation and could be affected by the growing conditions and the genotype characterized by different plant morphology, color of leaves, and chemotypes [3,4,5,6]. There is a strong correlation between the agronomic conditions and the profile and quantity of chemical compounds and further bioactive properties, starting with the genotype, climatic conditions, soil conditions, and agronomic techniques [7].

Nowadays, the pressure to improve the nutritional parameters and the bioactive quality of food products is increasing alongside with the environmental and economic concerns for the agroindustry sector. In order to fulfill these major requirements, it is necessary to find profitable, sustainable, and ecological nutritional solutions [8,9,10]. In this sense, soil fertilization for plant cultivation is a common practice that is used to achieve and sustain food production in order to feed the increasing human population and their increasing per capita food consumption.

Nitrogen (N) is the primary macronutrient affecting the physiology, yield, and quality of leafy crops, including basil [11,12,13,14,15]. Nevertheless, the common application of N for enhanced crop yields has multiple implications on aspects such as the economy, the quality and the environment, therefore modern agriculture requires rational N management [3,4]. N has some negative consequences on the environment when misused, at the same time there are also economic implications that may affect crop productivity and the quality of the final product [16,17]. The application of fertilizer doses exceeding the crop needs may generate a situation where nitrates accumulate in the soil causing plant luxury consumption and low N use efficiency [11,18,19], while the excess of nitrates may be leached out of the root zone and contaminate the aquifer [16,17]. The common application worldwide of soluble N fertilizer via the irrigation water (fertigation) with frequent delivery of small N doses directly in the root zone has the potential to improve the matching between fertilizer application and crop N demand, thereby minimizing both risks of economic loss and environmental pollution [20]. However, especially for aromatic crops like basil, even when using fertigation there is a need for defining the optimal N application rate considering not only agronomic performance but also the effects on chemical profile and bioactive properties of the crop.

Basil cultivation is conducted in open fields as well as under protected environments in both soil and soilless cultivation systems. Aiming to improve crop yield and to increase availability of basil leaves throughout the year, controlled cultivation in protective structures such as greenhouses is more appropriate than cultivation in an open field [16,17,21,22]. Previous studies where the N fertilization rate on basil plants was tested showed a positive effect on plant growth through a yield increase, although the higher crop production was not always accompanied with high quality for the final product [3,4]. In this context, the aim of this work was to evaluated the effect of different N rates (unfertilized control, 200, 400, and 600 ppm) on the nutritional parameters and the chemical profile of three red-colored basil cultivars: Red Basil, Dark Opal, and Basilico Rosso, and one green-colored landrace: Mitikas. In addition, the bioactive properties of leaves’ hydroalcoholic extracts were also examined in order to reveal how a common cultivation practice may affect the antioxidant and antimicrobial properties of basil leaves.

## 2. Materials and Methods

### 2.1. Samples and Sample Preparation

Seeds from three colored basil cultivars (*Ocimum basilicum* L.), namely Red Basil (Geniki Fytotechniki S.A., Athens, Greece), Dark Opal (De Corato Sementi, Andria, Italy), and Basilico Rosso (Larosa Emanuele Sementi, Andria, Italy) and one green local landrace (Mitikas) were sown in seed trays containing peat (Klassman-Deilmann, Tray Substrate, Geeste, Germany) on 04 April, 2019. Young seedlings were transferred at the stage of 3 or 4 true leaves in 2 L plastic pots containing peat (Klassman-Deilmann, KTS2, Geeste, Germany) and perlite (1:1, *v*/*v*) on 23 April, 2019. Four nitrogen fertilization rates were applied, namely Control (0 ppm N), 200 ppm, 400 ppm, and 600 ppm of nitrogen. Plants were fertigated with similar amount of 50–300 mL per pot, depending on the conditions. For each treatment, 15 pots were used with one plant per pot (60 pots in total).

Harvest took place on 14 June, 2019 and just before flower formation. Plants were harvested with scissors at the substrate level. After harvest, the aerial parts of the plants were weighed in order to estimate total fresh weight per plant and samples of fresh leaves (after removing shoots) from each treatment were pooled in batch samples and lyophilized, ground to powder, and put at −80 °C for chemical analyses. Using fresh yield data and applied N fertilizer rates, the N use efficiency index was calculated according to Di Gioia et al. [11].

The partial factor productivity of applied N (PFPN), which represents the g of product harvested per g of applied N was calculated as

PFPN = YF/NF(1)

The agronomic efficiency of applied N (AEN), which represents the g of yield increase per g of applied N was calculated as

AEN = (YF − Y0)/NF(2)

where YF is the crop yield (g/plant fresh weight) obtained with the application of a determinate N-fertilizer (NF) rate (g/plant); Y0 is the crop yield obtained in the unfertilized control.

Hunter color parameters (L∗, a∗ and b∗) were measured on the blades of the upper surface of leaves of the three red colored genotypes using a chroma meter (Chroma Meter CR400, Konica Minolta, Tokyo, Japan). Chroma (C*: relative saturation) and hue angle values (h°) were calculated according to the formulas previously described by the authors [23] following CIELab color space readings (L*, a* and b* values) that were measured through the computerized system.

### 2.2. Nutritional Value and Energy Content

Lyophilized leaves were characterized for proximate constituents following the AOAC international methods [24], including proteins (AOAC 978.04), crude fat (AOAC 920.85), ash (AOAC 923.03), and carbohydrates (calculated by difference) and expressed as g per 100 g of dried weight (dw). The energy (kcal per 100 g of dw) was determined based on regulation (EU) no. 1169/2011.

### 2.3. Chemical Characterization

#### 2.3.1. Organic Acids

Were determined by ultra-fast liquid chromatography (Shimadzu 20A series UFLC, Shimadzu Corporation, Kyoto, Japan) coupled to a diode-array detector operating in the optimized conditions described in detail by the authors [25]. The detected organic acids were identified following the comparison of their retention time and UV–vis spectra with commercial standards, while quantification was performed by using calibration curves. Data were obtained and evaluated with LabSolutions Multi LC-PDA software (Shimadzu Corporation, Kyoto, Japan).

#### 2.3.2. Free Sugars

Free sugars were determined with the implementation of a high-performance liquid chromatography (HPLC) system coupled to a refractive index detector and by applying the internal standard method (IS, melezitose; Sigma, St. Louis, MO, USA) [26]. Data were obtained and evaluated with Clarity 2.4 software (Informer Technologies, Inc., Solihull, UK).

#### 2.3.3. Tocopherols

Tocopherols were analyzed using the HPLC system coupled to a fluorescence detector, while tocol was used as IS [26]. Data were obtained and evaluated with Clarity 2.4 software (Informer Technologies, Inc., Solihull, UK).

#### 2.3.4. Fatty Acids

The profile of fatty acids was evaluated by gas-liquid chromatography (YOUNG IN Chromass 6500 GC System; YL Instruments, Anyang, Korea) coupled to a flame ionization detector (FID) [26]. Data were recorded and processed with Clarity 4.0 software (Informer Technologies, Inc., Solihull, UK).

### 2.4. Hydroethanolic Extracts Preparation

#### Extract Preparation

Hydroethanolic extracts were obtained after mixing 2.5 g of lyophilized sample with ethanol/water solution (80:20, *v*/*v*; 30 mL) and stirring for 60 min at room temperature. After filtering the supernatant (Whatman filter paper no. 4), the residue was re-extracted and the filtrates from both extractions were combined and concentrated under rotary evaporator at 40 °C and then lyophilized [26].

### 2.5. Phenolic Compound Determination

The abovementioned hydroethanolic extracts were redissolved in ethanol/water (80:20, *v*/*v*) to achieve the final concentration of 10 mg/mL. Extracts were analyzed in a HPLC system coupled with a diode-array detector (DAD) and a linear ion trap (LTQ XL) mass spectrometer (MS) equipped with an electrospray ionization (ESI) source. Phenolic compounds were separated in a Waters Spherisorb S3 ODS-2 C18 column (Waters Corporation, Milford, MA, USA). The operating conditions and the identification and quantification of the detected compounds was performed according to the protocol of Bessada et al. [27].

### 2.6. Bioactive Property Evaluation

#### 2.6.1. Antioxidant Activity

Bioactive properties were evaluated following two ex-vivo procedures: (a) the thiobarbituric acid reactive substances (TBARS) assay where porcine brain cell tissues obtained from local slaughterhouses (Bragança, Portugal) were used as oxidizable substrates [26], (b) the oxidative hemolysis (OxHLIA) assay evaluated for Δt of 60 and 120 min [26].

#### 2.6.2. Antimicrobial Properties

The antibacterial and antifungal properties of the extracts were evaluated with the microdilution method [28]. The positive controls used were E211 and E 224, whereas the negative control was 5% DMSO.

### 2.7. Statistical Analysis

The experimental procedure was carried out based on the randomized compete block (RCB) design with three repetitions. Crop performance and color parameters were evaluated in 15 individual plants (*n* = 15). For chemical analyses, three pooled samples were prepared for each treatment and each assay was performed in triplicate (*n* = 3). All the data were subjected to two-way ANOVA considering as factors the cultivars and the N concentration, while means were compared according to Tukey’s HSD test (*p* = 0.05). Quadratic regression analysis was performed using PROC REG of SAS software package (SAS Institute Inc., Cary, NC, USA) to estimate the parameters of the relationship between leaves, stem, total above-ground plant fresh weight, and N input. All the other analyses were performed with the statistical package SPSS v. 23.0 (IBM Corp., Armonk, NY, USA).

## 3. Results and Discussion

### 3.1. Effect of Genotype and Nitrogen Input on Basil Yield and Leaf Color

**Figure 1:**
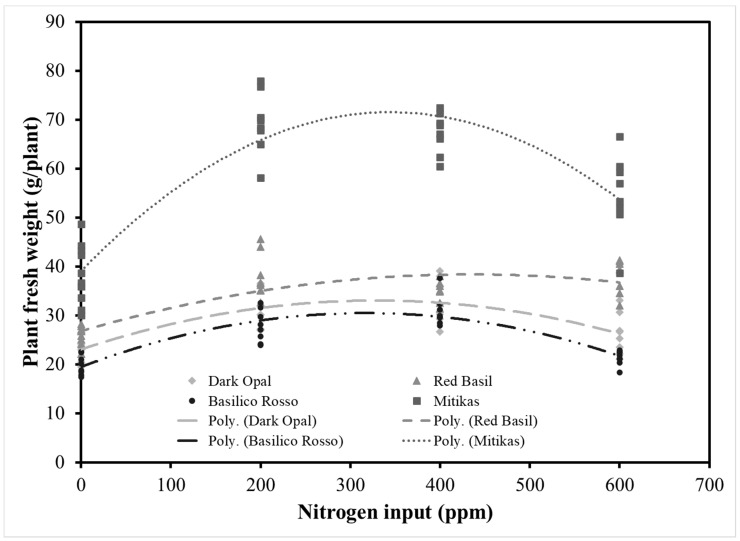
Above-ground plant fresh weight in response to nitrogen input in Dark Opal, Red Basil, Basilico Rosso, and Mitikas. Parameters of the quadratic response curve and their significance are presented in Table S1.

**Figure 2:**
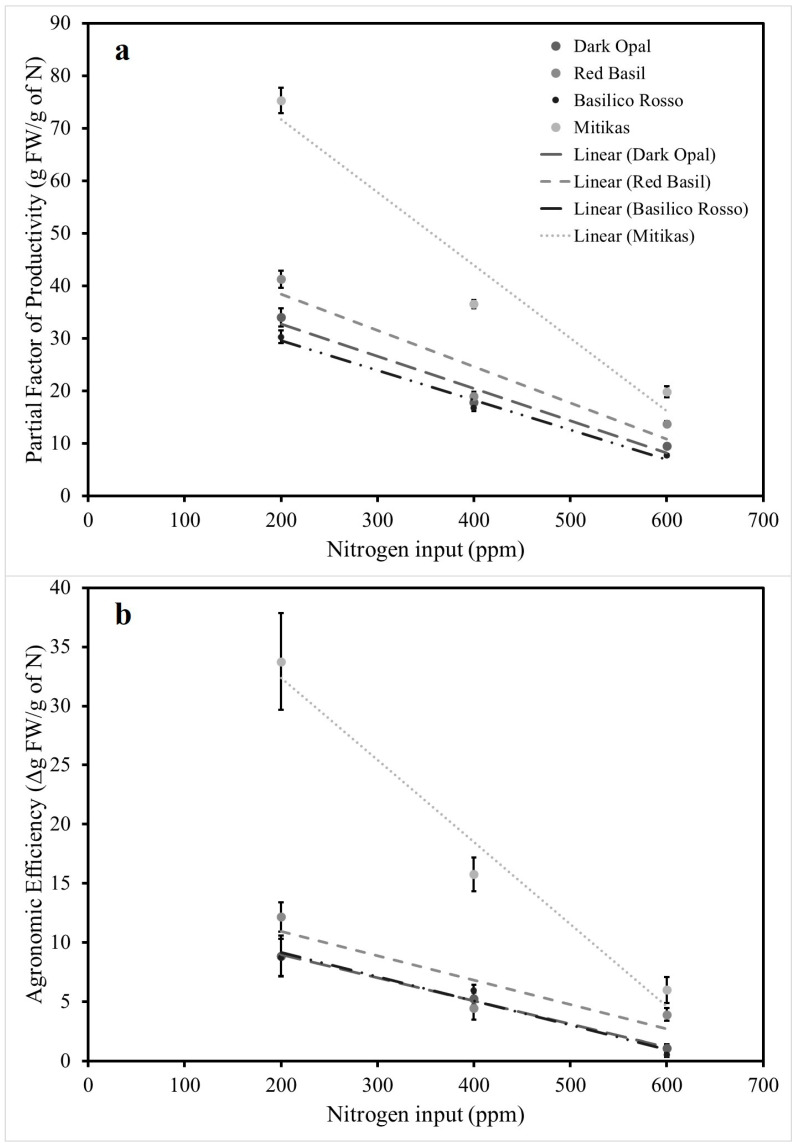
Effect of nitrogen input on (**a**) partial factor of productivity and (**b**) agronomic efficiency of applied nitrogen fertilizer in Dark Opal, Red Basil, Basilico Rosso, and Mitikas Basil.

**Figure 3:**
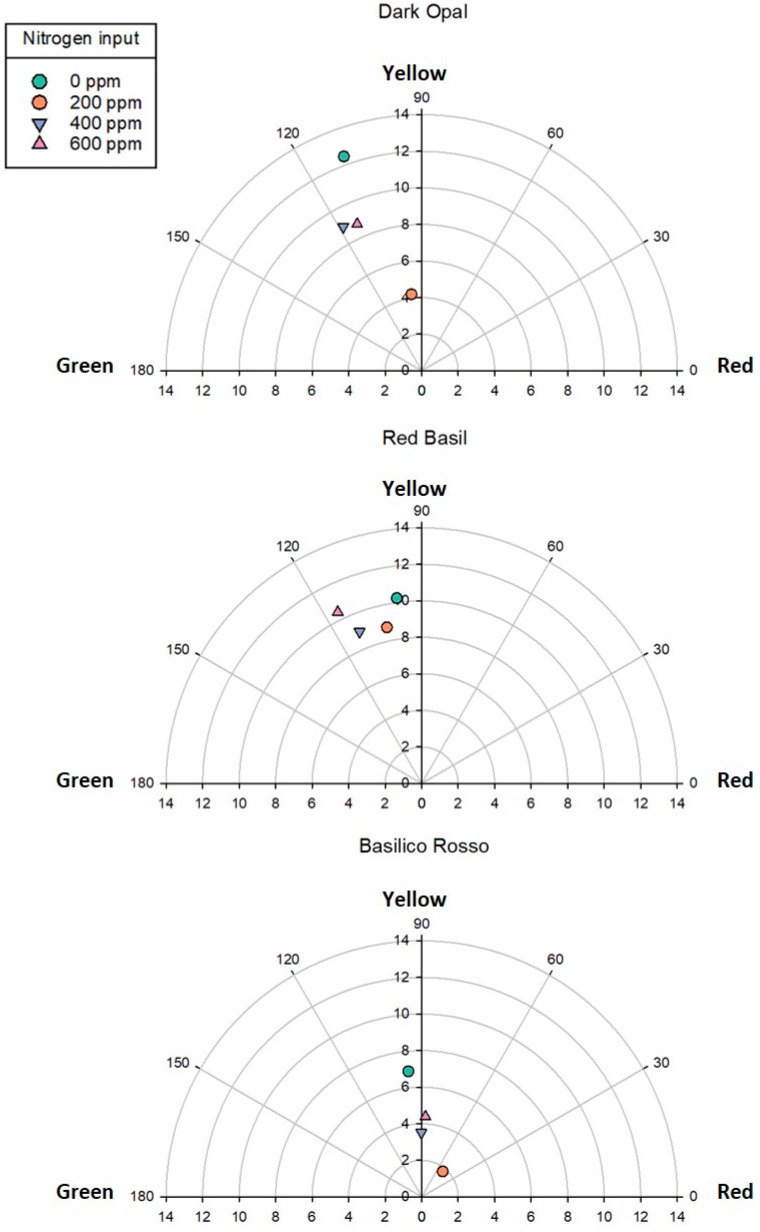
Chroma-hue plot of Dark Opal, Red Basil, and Basilico Rosso leaves in response to nitrogen input.

Basil fresh yield was significantly affected by both genotype and N input. In all four genotypes examined, leaf and total above-ground plant fresh biomass responded to N inputs according to a second order polynomial function (Figure 1). Estimated intercept, slope, and quadratic coefficient of the relationships examined were all significant except for the stem fresh weight—N input functions for which slope and quadratic coefficient of Dark Opal and Basilico Rosso were not significant (Table S1). The adjusted *R*^2^ (Adj *R*^2^) for the function describing the relationship between total above-ground fresh weight and N input was 0.50 in the case of Dark Opal and up to 0.79 in the case of Mitikas, while higher Adj *R*^2^ values were observed in the case of the functions describing the relationship between leaf fresh biomass and N input. Regardless the level of N input, Mitikas was the most productive genotype with a total above-ground plant fresh weight on average 68%, 102%, and 120% higher than Red Basil, Dark Opal, and Basilico Rosso. Similar results were reported by the authors [29] comparing Italian Classic green basil with Red Rubin basil grown in soilless system. Green basil response to N input was also characterized by a higher quadratic coefficient compared to the other varieties tested, suggesting a higher response to N inputs. Given the higher productivity, Mitikas nitrogen use efficiency was significantly higher compared to the three red genotypes examined. Consistently with previous studies [11,18], a decline of the nitrogen use efficiency was observed in all four genotypes with increasing the amount of N applied both in terms of fresh yield per unit of fertilizer (PFP_N_) and in terms of fresh yield increment per unit of fertilizer applied (AE_N_) (Figure 2). The level of N input influenced the leaf color of the three red genotypes which consistently showed higher lightness and chroma or color saturation when not fertilized compared to fertilized plants, and especially to those fertilized with 200 ppm of N, while little hue variations were observed (Figure 3).

### 3.2. Effect of Genotype and Nitrogen Input on Basil Leaves Nutritional Value

A significant interaction effect was recorded between genotype and N input on all the parameters defining the nutritional value (Table 1). On average, all four genotypes of basil examined had similar fat content (1.9 g/100 g dw); however, N input had a significant impact, and fat content was generally the highest in the unfertilized control and the lowest in plants fertigated with 200 ppm of N. The range of protein content was between 24.8 and 63.6 g/100 g dw and increased with increasing the level of N, although limited or no differences were observed between plants nourished with 200 and 400 ppm of N in the case of Red Basil and Basilico Rosso cultivars. Unfertilized plants consistently had the lowest level of proteins. Ash content ranged between 12.2 and 14.2 g/100 g dw and varied among the tested genotypes and N input levels with no consistent trend. Carbohydrates content varied between 21.4 and 60.1 g/100 g dw and was consistently the lowest in plants receiving 600 ppm of N, and the highest in plants not receiving fertilizer. The energetic value ranging from 351 to 362 kcal/100 g was influenced by both genotype and N input but with no consistent trends being observed. In agreement with previous studies that examined the nutritional value of basil leaves, in this study protein and carbohydrates were the major macronutrients [30,31], although higher levels of proteins were observed in this study in plants nourished with higher levels of N. Examining the nutritional value of dry green basil, Pereira et al. [32] observed similar levels of ash, but substantially higher levels of carbohydrates and lower levels of proteins compared to the present study. Such differences may be associated with different drying methods [33], different environmental conditions, and particularly to a lower level of N supply during cultivation and to genotypic differences [34,35,36].

### 3.3. Effect of Genotype and Nitrogen Input on Basil Leaves’ Organic Acids Content

The main organic acids identified were quinic, oxalic, and shikimic acid, while ascorbic acid was detected only in traces (Table 1). Fernandes et al. [30] reported a different organic acids profile in *O. basilicum* var. *purpurascens* leaves since they also detected malic, citric, fumaric, and ascorbic acid; however, consistently with this study, quinic acid was the primary compound. The leaves of the three red basil genotypes had similar levels of quinic acid ranging from 9 to 11 g/100 g dw, while Mitikas quinic acid content was on average 6 g/100 g dw. Oxalic acid concentration was the highest in Dark Opal, while the other three genotypes had similar content of oxalic acid. Total organic acid content was significantly greater in all three red basil genotypes compared to the green one. When examining the impact of the N input, it was observed that the concentration of each organic acid and the total organic acids content increased with increasing the level of N input in all four genotypes. These findings propose that the deficiency of N in unfertilized plants limited the synthesis of organic acids while the same plants accelerated the accumulation of carbohydrates (Table 1). According to other reports, nitrogen fertilization may affect organic acid composition and content with increasing nitrogen rates, resulting in high organic acids content [12,35,37].

### 3.4. Effect of Genotype and Nitrogen Input on Basil Leaves’ Free Sugar Content

Examining the profile of free sugars in basil leaves, a significant interaction was recorded for the genotype and N input effects (Table 2). Three soluble sugars were detected: glucose, fructose, and sucrose, whereas trehalose which was referred in previous studies [30,32] was not detected in this case. Glucose was the main sugar detected (3.4 g/100 g dw on average), followed by fructose (1.5 g/100 g dw on average) and sucrose (0.95 g/100 g dw on average) for all the genotypes with the exception of Basilico Rosso that regardless the level of N input had the highest concentration of sucrose (2.2 g/100 g dw on average) and a relatively lower content of fructose (0.9 g/100 g dw on average) compared to the other genotypes tested. Unfertilized plants were characterized by relatively lower content of glucose and higher proportion of fructose in all the genotypes except for Basilico Rosso. The highest concentrations of total free sugars were consistently observed in plants fertigated with the highest level of N input. Except for the absence of trehalose, these findings are in agreement with the ones of previous reports [30,32] and suggest that, in all the tested genotypes, the higher levels of N input are associated with enhanced content of total free sugars.

### 3.5. Effect of Genotype and Nitrogen Input on Basil Leaves’ Tocopherol Content

The tocopherols profile was influenced by the interactive effect of genotype and level of N input (Table 2). In all four genotypes the isomers α-, γ-, and δ-tocopherol were identified. The prevailing isomer was α-tocopherol followed by γ- and δ-tocopherol, respectively. *A*-tocopherol content ranged on average from a minimum of 0.8 mg/100 g dw in Basilico Rosso up to 4 mg/100 dw in Dark Opal and Red Basil. Consistently across the genotypes, the content of *α*-tocopherol was the highest in plants fertilized with 200 ppm of N, while further increase of N input resulted in a decrease of α-tocopherol. On average, the content of γ- and δ-tocopherol was almost equivalent within each genotype with the highest values observed for Dark Opal basil and the lowest content observed in the green basil. The tocopherol profile observed in this study was similar to the profile observed by the authors [38] in green basil, while did not match with the profile reported by the authors [30] who found all four vitamers of vitamin E (α-, β-, γ-, and δ-tocopherol) in the leaves of *O. basilicum* var. *purpurascens*, with γ-tocopherol prevailing over the other isomers. The range of total tocopherols content was between 1.3 and 9.34 mg/100 g dw, and was consistently the highest in the leaves of plants fertigated with 200 ppm of N, followed by those fertigated with 400 ppm of N or non-fertilized, whereas the lowest levels of total tocopherols were generally observed in plants fertigated with the highest level of N input tested. The proportion between different tocopherol isomers and their relative content seems to be primarily determined by the basil genotype. However, the large variation of tocopherol content within the same genotype as a function of the level of N supply suggests that the availability of N plays a pivotal role in determining the absolute content of tocopherols, and both deficiency and excess of N seem to negatively affect the content of vitamin E.

### 3.6. Effect of Genotype and Nitrogen Input on Basil Leaves’ Fatty Acids Content

A total of fifteen fatty acids were detected in all four basil genotypes tested (Table S2). The main fatty acids were *α*-linolenic acid (omega-3 fatty acid) and linoleic acid (omega-6 fatty acid) among the polyunsaturated fatty acids (PUFA) and palmitic acid among the saturated fatty acids (SFA) (Table 3). Alpha-linolenic acid, the most abundant fatty acid ranged from 31.3% up to 51% of the total fatty acid content, and it was on average higher in Red Basil, followed by Basilico Rosso and Dark Opal and significantly lower in the green basil leaves. Examining the proportion of the fatty acids categories, consistently with the findings found by Fernandes et al. [30] in *O. basilicum* var. *purpurascens,* in the present study PUFAs constituted the main category of fatty acids in all four genotypes. PUFAs percentage was the highest in Red Basil and the lowest in Mitikas, which instead had the highest proportion of MUFAs, while Red Basil had the lowest level of SFA. Examining the *n*-6/*n*-3 PUFAs ratio, the lowest ratio which indicates a high nutritional value was observed in Red Basil (0.29) and the highest in the leaves of green basil (0.33). The effect of N input on the fatty acid profile varied with the cultivar. The *n*-6/*n*-3 PUFAs ratio was affected by N input and was lower in the leaves of plants fertigated with 200 ppm of N, with the exception of Dark Opal that had the lowest *n*-6/*n*-3 PUFAs ratio at 400 ppm of N and the highest at 200 ppm of N. Overall, these results confirm that basil, and particularly red genotypes, have an interesting fatty acid profile characterized by low *n*-6/*n*-3 ratio and by high content of *α*-linolenic acid (*n*-3) which has several potential beneficial effects on human health [39,40].

### 3.7. Effect of Genotype and Nitrogen Input on Basil Leaves’ Hydroethanolic Extract Phenolic Content

The results regarding the identification and quantification of polyphenols are presented in Table 4 and Table 5, respectively. Basil genotype and the level of N input had a significant interactive effect on the profile of phenolic compounds. In all four genotypes were detected six phenolic compounds, including four phenolic acids: caffeic and chicoric acid (hydroxycinnamic acids), sagerinic acid, and salvianolic acid F, and two flavonoids: quercetin and eriodictyol, a flavonol and a flavanone derivate, respectively. Sagerinic acid was the main phenolic compound detected followed by the eriodictyol derivate and by salvianolic acid F. These findings are in contrast with the findings of previous reports where rosmarinic acid and/or chicoric acid were the main phenolic compounds detected depending on the basil cultivar examined [13,41,42,43]. Nevertheless, the present work and previous studies suggest that the genotype plays an important role in determining the specific phenolic profile. In fact, large variations of the content of the main phenolic compounds were observed between the four basil cultivars tested that further reinforces this argument. Sagerinic acid ranged on average from 3 mg/g of extract in the green basil up to 11 mg/g of extract in Dark Opal and Red Basil. The content of eriodictyol derivate ranged between 1 mg/g of extract in Mitikas and 7 mg/g of extract in Red Basil.

Red Basil and Dark Opal basil had the highest content of total phenolic acids and total flavonoids, and thus had the highest level of total phenolic compounds (25 mg/g of extract on average) whereas the green basil had the lowest content of total phenolic compounds (on average 9 mg/g of extract). These findings are consistent with the report by Nguyen and Niemeyer [13] who observed substantially lower levels of phenolic compounds in Genovese green basil as compared to Dark Opal basil. Large variations of both phenolic acids and flavonoid derivates content were observed also in function of the level of N input. In Dark Opal basil for example, the concentration of sagerinic acid ranged from a maximum of 24.6 mg/g of extract in unfertilized plants to a minimum of 4.6 mg/g of extract in plants fertigated with 600 ppm of N. In all four basil genotypes examined, the content of total phenolic acids, total flavonoids and total phenolic compounds consistently decreased with increasing the level of N input, suggesting that in presence of higher levels of N enhanced plant growth may be inhibiting the biosynthesis of phenolic compounds, while the stress associated with the deficiency of N could have activated the biosynthesis of phenolic compounds while negatively impacted plant growth. These results are in agreement with the findings of Nguyen and Niemeyer [13] who examining the phenolic profile of Sweet Thai, Dark Opal, and Genovese basil in response to increasing N levels observed significantly higher levels of phenolic compounds and especially of rosmarinic acid at the lower levels of N in Dark Opal and Genovese basil. Similarly, Prinsi et al. [29] recorded an increase of rosmarinic and total phenolic acids content in green and red basil grown in a hydroponic system after N starvation for 5 days before harvest.

### 3.8. Effect of Genotype and Nitrogen Input on Basil Leaves’ Hydroethanolic Extract Bioactive Properties

The antioxidant capacity of the basil leaves hydroethanolic extract was evaluated through two cell-based methods, namely the TBARS method which measures the lipid peroxidation inhibition and the OxHLIA method which estimates the capacity of the extract in delaying oxidative hemolysis in sheep erythrocytes (Table 6). With the TBARS assay, it was observed that leaves of Dark Opal basil fertigated with 200 ppm of N had the lowest EC_50_ value (13.1 μg/mL) and thus the highest antioxidant activity, followed by leaves of Red Basil (25.2 μg/mL) and Basilico Rosso (31 μg/mL) grown with 200 ppm of N. Instead, green basil and Red Basil grown with the highest levels of N had the highest EC_50_ and thus the lowest antioxidant activity. Even using the OxHLIA assay, the lowest half maximal inhibitory concentrations (IC_50_) calculated after one or two hours were observed in the extract of Dark Opal and Red Basil fertigated with 200 ppm of N, whereas the highest values were observed for the extract of Mitikas grown with the highest level of N. The level of antioxidant activity resulting from the interaction between the basil genotypes and the level of N input tested was potentially positively correlated to the total tocopherol content which was the highest in Dark Opal and Red Basil fertigated with 200 ppm and the lowest in Basilico Rosso and green basil fertigated with the highest levels of N (Table 2). Similar results were reported by the authors [44] who also suggested significant differences between two *Ocimum* species (*O. basilicum* cv. Cinnamon and *O. citriodorum*) and they recorded IC_50_ values significantly lower than the positive control (Trolox). Moreover, Sikora et al. [45] highlighted the antiradical properties of basil leaves extracts against post-processing browning of shredded lettuce due to the existence of easily bioaccessible polyphenols.

Examining the activity of the basil leaves’ hydroethanolic extracts against bacteria, the lowest MIC (minimum inhibiting concentration) and MBC (minimum bactericidal concentration) values and thus the highest antibacterial activity were observed against *B. cereus* with specific treatments having values lower than the positive controls E211 and E224 (Table 7). Values of MIC and MBC similar or below the positive controls were observed also for *Staphylococcus aureus* and *Salmonella tymphimurium*, but not a particular trend was observed in the potential antibacterial activity of a specific genotype and/or level of N input. Examining the antifungal activity of the basil leaves’ extracts it was observed that selected treatments exhibited lower values MIC and MFC (minimum fungicidal concentration) values than *Aspergillus fumigatus*, *A. versicolor*, and *Trichoderma viride* (Table 8). Moreover, specific extracts had lower MIC and similar MFC (minimum fungicidal concentration) values to those recorded for the two positive controls against *A. niger*, *Penicillium funiculosum*, *P. verrucosum var. cyclopium*, and *A. fumigatus*. Similarly to our study, Fernandes et al. [30] reported significant antibacterial activities for *Ocimum basilicum* var. *purpurascens* leaves; extracts which exhibited lower MIC, MBC, and MFC values than the used positive controls. However, these findings cannot be compared with the results of our study since different genotypes and positive controls were tested.

## 4. Conclusions

Finding the right balance between yield, nutritional value, and bioactivity in aromatic vegetables like basil could be challenging because of the complex interaction between genotype and environmental conditions. The present study contributes to significantly advance our knowledge of such complex interaction by examining the effects of N input, one of the most important nutrients, on the yield, chemical profile, and leaf extract bioactivity of one green- and three red-colored genotypes. The study revealed a significant interactive impact of both factors. While green basil was more productive, red-colored leaves had a higher nutritional and bioactive profile showing a higher content of α-, γ-, δ-, and total tocopherols, a higher proportion of PUFAs and a lower n-6/n-3 PUFAs ratio, along with a higher content of total flavonoids and total phenolic compounds, which resulted in a higher antioxidant capacity of the leaf hydroethanolic extracts. However, the chemical profile of each basil genotype was modulated by the level of N input, and while the concentration of total phenolic compounds decreased with the increase of N input the content of α-, γ-, δ-, and total tocopherols was enhanced in plants fertigated with 200 ppm of N and decreased in those receiving 600 ppm of N. Similarly, the fatty acid and PUFA content were enhanced in plants fertigated with 200 ppm of N which showed the lowest n-6/n-3 PUFAs ratio in three out of four basil cultivar tested, with the exception of Dark Opal basil that had the best fatty acid profile with the lowest n-6/n-3 PUFAs ratio when fertigated with 400 ppm of N. Plants fertigated with 200 ppm of N had also the highest antioxidant activity particularly in Dark Opal and Red Basil leaf extracts, whereas all genotypes showed good antibacterial and antifungal activity, regardless the level of N input. Further studies are warranted to examine larger set of genotypes and other environmental and cultivation factors that may have a significant impact on the chemical profile and bioactive effects of this important aromatic crop.

## Figures and Tables

**Table 1:** Nutritional value (g/100 g dw), energetic value (kcal/100 g dw) and organic acids (g/100 g dw) of the studied basil genotypes in relation to nitrogen input (mean ± SD, *n* = 3).

Cultivar	ppm	Nutritional Value				
		Fat	Proteins	Ash	Carbohydrates	Energy
Dark Opal	0	1.9 ± 0.2 ^A,^*	**47 ± 14 ^A^**	**12.9 ± 0.2 ^B^**	**38 ± 14 ^B^**	**358 ± 1 ^A^**
		2.17 ± 0.01 ^a,b^	26 ± 2 ^h^	12.8 ± 0.1 ^h^	59 ± 1 ^b^	359.8 ± 0.2 ^b^
	200	1.69 ± 0.01 ^g^	46.8 ± 0.2 ^g^	12.8 ± 0.3 ^h^	38.71 ± 0.08 ^c^	357 ± 1 ^e,f,g^
	400	1.79 ± 0.02 ^e^	51.1 ± 0.6 ^d,e^	13.1 ± 0.1 ^e,f^	34.0 ± 0.5 ^g^	356.5 ± 0.3 ^g,h^
	600	1.97 ± 0.03 ^d^	63.6 ± 0.6 ^a^	13.0 ± 0.3 ^f,g^	21.4 ± 0.7 ^j^	357.7 ± 0.8 ^e,f^
Red Basil	0	**1.9 ± 0.3 ^A^**	**45 ± 11 ^B^**	**13.0 ± 0.6 ^A^**	**40 ± 11 ^A^**	**357 ± 2 ^A^**
		2.19 ± 0.01 ^a,b^	25.4 ± 0.8 ^h,i^	12.9 ± 0.1 ^f,g,h^	59.5 ± 0.6 ^a,b^	359.3 ± 0.3 ^b,c^
	200	1.40 ± 0.02 ^i^	51.5 ± 0.5 ^d^	12.1 ± 0.2 ^i^	35.0 ± 0.2 ^d,e,f^	358.7 ± 0.7 ^c,d^
	400	1.78 ± 0.01 ^e,f^	50.4 ± 0.8 ^e^	13.6 ± 0.5 ^c^	34.3 ± 0.2 ^f,g^	355 ± 1 ^i^
	600	2.06 ± 0.07 ^c^	52.3 ± 0.1 ^c^	13.3 ± 0.1 ^d,e^	32.3 ± 0.1 ^h^	356.9 ± 0.4 ^f,g^
Basilico Rosso	0	**1.9 ± 0.2 ^A^**	**45 ± 11 ^B^**	**12.7 ± 0.6 ^A,B^**	**40 ± 12 ^A^**	**359 ± 3 ^A^**
		2.17 ± 0.02 ^b^	25.5 ± 0.9 ^h,i^	12.2 ± 0.4 ^i^	60.1 ± 0.9 ^a^	362 ± 1 ^a^
	200	1.58 ± 0.03 ^h^	51.08 ± 0.06 ^d,e^	12.2 ± 0.3 ^i^	35.1 ± 0.3 ^d,e^	359.0 ± 0.8 ^b,c,d^
	400	1.79 ± 0.04 ^e,f^	50.6 ± 0.7 ^e^	13.4 ± 0.2 ^c,d^	34.1 ± 0.6 ^g^	355.2 ± 0.7 ^i^
	600	2.06 ± 0.01 ^c^	52.9 ± 0.9 ^c^	13.0 ± 0.1 ^f,g^	32.0 ± 0.6 ^h^	358.2 ± 0.1 ^d,e^
Mitikas	0	**1.9 ± 0.2 ^A^**	**46 ± 14 ^A^**	**13.6 ± 0.5 ^A^**	**38 ± 13 ^B^**	**355 ± 3 ^B^**
		2.21 ± 0.05 ^a^	24.8 ± 0.4 ^i^	13.9 ± 0.1 ^b^	59.1 ± 0.4 ^b^	355.5 ± 0.1 ^h,i^
	200	1.60 ± 0.07 ^h^	48.9 ± 0.6 ^f^	14.2 ± 0.2 ^a^	35.3 ± 0.5 ^d^	351.0 ± 0.8 ^j^
	400	1.75 ± 0.03 ^f^	50.4 ± 0.8 ^e^	13.4 ± 0.3 ^c,d^	34.4 ± 0.8 ^e,f,g^	355.0 ± 0.8 ^i^
	600	2.08 ± 0.01 ^c^	61.5 ± 0.8 ^b^	12.86 ± 0.04 ^g,h^	23.5 ± 0.5 ^i^	358.9 ± 0.1 ^b,c,d^
**Cultivar**	**ppm**	**Organic acids**				
		**Oxalic acid**	**Quinic acid**	**Shikimic acid**	**Ascorbic acid**	**Total organic acids**
Dark Opal	0	5.7 ± 0.8 ^A,^*	**9 ± 3 ^B^**	**0.11 ± 0.01 ^A^**		**15 ± 4 ^A,B^**
		4.67 ± 0.01 ^i^	4.45 ± 0.01 ^n^	0.120 ± 0.001 ^b^	tr	9.24 ± 0.01 ^m^
	200	5.27 ± 0.05 ^h^	9.80 ± 0.08 ^h^	0.100 ± 0.003 ^e,f^	tr	15.18 ± 0.02 ^i^
	400	6.01 ± 0.09 ^c,d^	10.57 ± 0.06 ^f^	0.100 ± 0.001 ^e^	tr	16.68 ± 0.03 ^g^
	600	6.78 ± 0.07 ^a^	12.41 ± 0.02 ^b^	0.100 ± 0.001 ^e,f^	tr	19.29 ± 0.05 ^b^
Red Basil	0	**5 ± 1 ^B^**	**10 ± 3 ^A,B^**	**0.09 ± 0.04 ^B^**		**15 ± 4 ^A,B^**
		3.17 ± 0.01 ^j^	4.50 ± 0.03 ^m^	0.020 ± 0.001 ^j^	tr	7.69 ± 0.03 ^n^
	200	5.33 ± 0.02 ^g^	10.37 ± 0.02 ^g^	0.100 ± 0.007 ^f^	tr	15.80 ± 0.05 ^h^
	400	5.76 ± 0.06 ^f^	11.07 ± 0.02 ^e^	0.110 ± 0.001 ^d^	tr	16.94 ± 0.04 ^f^
	600	6.04 ± 0.01 ^c^	12.41 ± 0.04 ^b^	0.130 ± 0.001 ^a^	tr	18.59 ± 0.05 ^c^
Basilico Rosso	0	**5 ± 2 ^B^**	**11 ± 4 ^A^**	**0.09 ± 0.04 ^B^**		**16 ± 5 ^A^**
		2.41 ± 0.01 ^l^	4.95 ± 0.06 ^l^	0.030 ± 0.001 ^i^	tr	7.39 ± 0.06 ^o^
	200	5.97 ± 0.01 ^d,e^	11.40 ± 0.02 ^d^	0.110 ± 0.002 ^c^	tr	17.48 ± 0.02 ^e^
	400	6.31 ± 0.09 ^b^	11.50 ± 0.05 ^c^	0.110 ± 0.001 ^c,d^	tr	17.92 ± 0.04 ^d^
	600	6.78 ± 0.01 ^a^	14.90 ± 0.06 ^a^	0.120 ± 0.001 ^b^	tr	21.80 ± 0.07 ^a^
Mitikas	0	**5 ± 1 ^B^**	**6 ± 1 ^C^**	**0.04 ± 0.02 ^C^**		**12 ± 3 ^C^**
		2.90 ± 0.02 ^k^	4.14 ± 0.04 ^o^	0.020 ± 0.001 ^j^	tr	7.06 ± 0.01 ^p^
	200	5.25 ± 0.01 ^h^	6.81 ± 0.02 ^k^	0.030 ± 0.001 ^i^	tr	12.09 ± 0.02 ^l^
	400	5.77 ± 0.03 ^f^	7.14 ± 0.02 ^j^	0.060 ± 0.003 ^h^	tr	12.97 ± 0.02 ^k^
	600	5.95 ± 0.07 ^e^	7.89 ± 0.01 ^i^	0.070 ± 0.003 ^g^	tr	13.92 ± 0.09 ^j^

tr–traces; * Different capital letters (A–C) within each column represent significant differences between the means of the four types of cultivars. Different small letters (a–p) within each column represent significant differences between the means of each level of nitrogen input and cultivar. In both cases, the Tukey’s HSD test was used at *p* = 0.05.

**Table 2:** Composition in free sugars (g/100 g dw) and tocopherols (mg/100 g dw) of the studied basil genotypes in relation to nitrogen input (mean ± SD, *n* = 3).

Cultivar	ppm	Free Sugars			
		Fructose	Glucose	Sucrose	Total Free Sugars
Dark Opal	0	1.7 ± 0.5 ^A,^*	**3 ± 1 ^C^**	**0.9 ± 0.2 ^B^**	**5 ± 2 ^C^**
		1.65 ± 0.04 ^f^	0.87 ± 0.01 ^l^	0.62 ± 0.01 ^i^	3.14 ± 0.06 ^j^
	200	0.92 ± 0.01 ^j^	2.51 ± 0.01 ^j^	0.95 ± 0.01 ^g^	4.38 ± 0.01 ^i^
	400	2.05 ± 0.09 ^b^	3.25 ± 0.06 ^h^	1.04 ± 0.05 ^f^	6.34 ± 0.07 ^f^
	600	2.32 ± 0.03 ^a^	3.45 ± 0.02 ^g^	1.07 ± 0.01 ^e^	6.84 ± 0.06 ^c^
Red Basil	0	**1.7 ± 0.4 ^A^**	**3 ± 1 ^C^**	**0.6 ± 0.2 ^C^**	**5 ± 1 ^B,C^**
		1.40 ± 0.02 ^h^	0.64 ± 0.02 ^m^	0.53 ± 0.05 ^j^	2.57 ± 0.09 ^k^
	200	1.55 ± 0.01 ^g^	2.69 ± 0.04 ^i^	0.878 ± 0.004 ^h^	5.11 ± 0.04 ^h^
	400	1.40 ± 0.05 ^h^	3.73 ± 0.01 ^f^	0.442 ± 0.006 ^k^	5.57 ± 0.05 ^g^
	600	2.29 ± 0.02 ^a^	3.76 ± 0.02 ^e^	0.51 ± 0.03 ^j^	6.57 ± 0.07 ^e^
Basilico Rosso	0	**0.9 ± 0.4 ^B^**	**3.5 ± 0.6 ^A,B^**	**2.2 ± 0.1 ^A^**	**7 ± 1 ^A^**
		0.55 ± 0.04 ^k^	2.48 ± 0.04 ^j^	2.03 ± 0.04 ^d^	5.06 ± 0.05 ^h^
	200	0.52 ± 0.02 ^k^	3.71 ± 0.03 ^f^	2.09 ± 0.03 ^c^	6.31 ± 0.02 ^f^
	400	1.08 ± 0.01 ^i^	3.96 ± 0.02 ^d^	2.17 ± 0.02 ^b^	7.21 ± 0.05 ^b^
	600	1.40 ± 0.05 ^h^	3.98 ± 0.01 ^d^	2.41 ± 0.01 ^a^	7.79 ± 0.04 ^a^
Mitikas	0	**1.8 ± 0.2 ^A^**	**4 ± 2 ^A^**	**0.11 ± 0.03 ^D^**	**6 ± 2 ^A,B^**
		1.52 ± 0.04 ^g^	0.95 ± 0.06 ^k^	0.096 ± 0.004 ^m^	2.57 ± 0.09 ^k^
	200	1.74 ± 0.05 ^e^	4.48 ± 0.05 ^c^	0.092 ± 0.003 ^m^	6.32 ± 0.01 ^f^
	400	1.85 ± 0.06 ^d^	4.82 ± 0.03 ^b^	0.109 ± 0.001 ^m^	6.78 ± 0.09 ^d^
	600	2.01 ± 0.06 ^c^	4.99 ± 0.01 ^a^	0.160 ± 0.004 ^l^	7.16 ± 0.05 ^b^
**Cultivar**	**ppm**	**Tocopherols**			
		***α*-Tocopherol**	***γ*-Tocopherol**	***δ*-Tocopherol**	**Total Tocopherols**
Dark Opal	0	4 ± 1 ^A,^*	**0.8 ± 0.3 ^A^**	**0.8 ± 0.3 ^A^**	**5 ± 2 ^A^**
		3.60 ± 0.03 ^d^	0.44 ± 0.03 ^f^	0.325 ± 0.008 ^h^	4.37 ± 0.05 ^d^
	200	6.07 ± 0.03 ^b^	1.32 ± 0.01 ^a^	1.18 ± 0.03 ^a^	8.58 ± 0.01 ^b^
	400	2.71 ± 0.01 ^g^	0.94 ± 0.02 ^c^	0.97 ± 0.02 ^c^	4.63 ± 0.01 ^c^
	600	2.41 ± 0.02 ^h^	0.60 ± 0.03 ^e^	0.76 ± 0.03 ^e^	3.77 ± 0.03 ^f^
Red Basil	0	**4 ± 2 ^A,B^**	**0.6 ± 0.3 ^B^**	**0.5 ± 0.4 ^B^**	**5 ± 3 ^A^**
		3.14 ± 0.02 ^e^	0.447 ± 0.001 ^f^	0.31 ± 0.02 ^h^	3.90 ± 0.01 ^e^
	200	7.09 ± 0.02 ^a^	1.13 ± 0.01 ^b^	1.12 ± 0.01 ^b^	9.34 ± 0.02 ^a^
	400	3.80 ± 0.03 ^c^	0.41 ± 0.01 ^g^	0.40 ± 0.02 ^f^	4.61 ± 0.01 ^c^
	600	1.71 ± 0.01 ^j^	0.340 ± 0.002 ^h^	0.259 ± 0.003 ^i^	2.31 ± 0.01 ^j^
Basilico Rosso	0	**0.8 ± 0.3 ^D^**	**0.5 ± 0.1 ^B,C^**	**0.5 ± 0.3 ^B^**	**1.8 ± 0.6 ^C^**
		0.55 ± 0.02 ^o^	0.44 ± 0.01 ^f^	0.313 ± 0.004 ^h^	1.30 ± 0.01 ^n^
	200	1.257 ± 0.001 ^l^	0.49 ± 0.02 ^e^	0.91 ± 0.04 ^d^	2.66 ± 0.01 ^i^
	400	0.87 ± 0.02 ^m^	0.67 ± 0.03 ^d^	0.370 ± 0.002 ^g^	1.92 ± 0.02 ^l^
	600	0.66 ± 0.02 ^n^	0.35 ± 0.04 ^h^	0.32 ± 0.02 ^h^	1.33 ± 0.08 ^n^
Mitikas	0	**1.9 ± 0.7 ^C^**	**0.36 ± 0.07 ^D^**	**0.27 ± 0.06 ^C^**	**2.6 ± 0.8 ^B^**
		2.36 ± 0.04 ^i^	0.445 ± 0.003 ^f^	0.315 ± 0.002 ^h^	3.13 ± 0.05 ^h^
	200	2.76 ± 0.05 ^f^	0.396 ± 0.004 ^g^	0.325 ± 0.001 ^h^	3.48 ± 0.04 ^g^
	400	1.55 ± 0.01 ^k^	0.321 ± 0.005 ^i^	0.255 ± 0.004 ^i^	2.12 ± 0.01 ^k^
	600	1.13 ± 0.01 ^m^	0.278 ± 0.004 ^j^	0.171 ± 0.002 ^j^	1.58 ± 0.01 ^m^

* Different capital letters (A–D) within each column represent significant differences between the means of the four types of cultivars. Different small letters (a–o) within each column represent significant differences between the means of each level of nitrogen input and cultivar. In both cases, the Tukey’s HSD test was used at *p* = 0.05.

**Table 3:** Main fatty acids and fatty acid groups (%) present in the hydroethanolic extracts of basil leaves in relation to nitrogen input (mean ± SD, *n* = 3).

Cultivar	ppm	Fatty Acids			Fatty Acid Groups			
		C16:0	C18:2n6c	C18:3n3	SFA	MUFA	PUFA	*n*6/*n*3
Dark Opal	0	20.8 ± 0.8 ^A,B,^*	**14.4 ± 0.7 ^A^**	**46 ± 3 ^B^**	**31 ± 2 ^A^**	**7.8 ± 0.4 ^B,C^**	**61 ± 2 ^B^**	**0.34 ± 0.01 ^A^**
		20.9 ± 0.2 ^d^	14.6 ± 0.4 ^c^	44.6 ± 0.3 ^i^	32.1 ± 0.1 ^c^	8.2 ± 0.1 ^g^	59.7 ± 0.2 ^h^	0.45 ± 0.01 ^a^
	200	21.8 ± 0.2 ^b^	15.2 ± 0.2 ^b^	41.8 ± 0.1 ^j^	34.1 ± 0.2 ^b^	8.2 ± 0.2 ^g^	57.8 ± 0.4 ^i^	0.36 ± 0.01 ^b^
	400	20.5 ± 0.7 ^e^	13.4 ± 0.4 ^i^	48.9 ± 0.2 ^d,e^	29.7 ± 0.6 ^d^	7.5 ± 0.1 ^h,i^	62.8 ± 0.6 ^e^	0.274 ± 0.006 ^j^
	600	19.87 ± 0.07 ^f^	14.4 ± 0.4 ^c,d,e^	49.08 ± 0.04 ^c,d^	28.8 ± 0.4 ^e,f^	7.3 ± 0.1 ^j^	64.0 ± 0.5 ^c^	0.292 ± 0.008 ^g,h^
Red Basil	0	**18.5 ± 0.6 ^C^**	**14.4 ± 0.4 ^A^**	**49.5 ± 0.6 ^A^**	**27.9 ± 0.8 ^C^**	**7.7 ± 0.5 ^B,C^**	**64.4 ± 0.7 ^A^**	**0.29 ± 0.01 ^B^**
		18.89 ± 0.05 ^h^	14.17 ± 0.07 ^e,f,g^	48.6 ± 0.4 ^e^	28.5 ± 0.2 ^f^	8.12 ± 0.1 ^g^	63.3 ± 0.4 ^d^	0.29 ± 0.01 ^h^
	200	19.4 ± 0.1 ^g^	14.06 ± 0.07 ^f,g^	50.03 ± 0.06 ^b^	28.5 ± 0.1 ^f^	7.0 ± 0.1 ^l^	64.5 ± 0.1 ^b^	0.281 ± 0.002 ^h,i^
	400	18.06 ± 0.01 ^i^	14.29 ± 0.06 ^d,e,f^	50.08 ± 0.07 ^b^	27.69 ± 0.02 ^g^	7.43 ± 0.04 ^i^	64.9 ± 0.1 ^a,b^	0.285 ± 0.002 ^h^
	600	17.78 ± 0.08 ^j^	15.13 ± 0.01 ^b^	49.3 ± 0.1 ^c^	26.7 ± 0.1 ^j^	8.31 ± 0.02 ^f^	65.0 ± 0.1 ^a^	0.307 ± 0.001 ^e^
Basilico Rosso	0	**20 ± 1 ^B^**	**14 ± 1 ^A^**	**47 ± 2 ^B^**	**29 ± 1 ^A,B^**	**8.3 ± 0.5 ^B^**	**62 ± 2 ^B^**	**0.30 ± 0.01 ^B^**
		21.5 ± 0.5 ^b,c^	15.5 ± 0.2 ^a^	45.4 ± 0.4 ^h^	30.0 ± 0.5 ^d^	8.8 ± 0.1 ^d^	61.2 ± 0.6 ^g^	0.341 ± 0.002 ^c^
	200	18.43 ± 0.01 ^i,j^	13.3 ± 0.1 ^i,n^	51.0 ± 0.2 ^a^	27.2 ± 0.1 ^h^	7.6 ± 0.1 ^h^	65.2 ± 0.1 ^a^	0.261 ± 0.002 ^k^
	400	20.5 ± 0.8 ^e^	15.2 ± 0.1 ^b^	45.5 ± 0.7 ^h^	29.8 ± 0.8 ^d^	8.5 ± 0.1 ^e^	61.7 ± 0.8 ^f^	0.33 ± 0.01 ^d^
	600	20.3 ± 0.6 ^e^	13.17 ± 0.05 ^i^	47.3 ± 0.5 ^f^	29.9 ± 0.6 ^d^	8.4 ± 0.1 ^f^	61.8 ± 0.6 ^f^	0.278 ± 0.002 ^j^
Mitikas	0	**22 ± 3 ^A^**	**13.9 ± 0.4 ^A,B^**	**43 ± 7 ^C^**	**30 ± 5 ^A^**	**12 ± 2 ^A^**	**57 ± 7 ^C^**	**0.33 ± 0.01 ^A^**
		27.36 ± 0.01 ^a^	13.4 ± 0.3 ^i^	31.3 ± 0.1 ^k^	38.5 ± 0.1 ^a^	16.2 ± 0.3 ^a^	45.3 ± 0.3 ^j^	0.43 ± 0.01 ^a^
	200	19.12 ± 0.06 ^g,h^	14.04 ± 0.57 ^g^	48.5 ± 0.5 ^e^	26.2 ± 0.1 ^i^	10.8 ± 0.1 ^c^	63.0 ± 0.1 ^d,e^	0.29 ± 0.01 ^h^
	400	19.8 ± 0.2 ^f^	14.45 ± 0.04 ^c,d^	46.5 ± 0.1 ^g^	27.3 ± 0.2 ^g,h^	11.3 ± 0.1 ^b^	61.4 ± 0.1 ^f,g^	0.311 ± 0.002 ^e^
	600	21.2 ± 0.6 ^c,d^	13.7 ± 0.2 ^h^	45.7 ± 0.6 ^h^	29.1 ± 0.6 ^e^	10.8 ± 0.2 ^c^	60.1 ± 0.8 ^h^	0.300 ± 0.001 ^f^

* Different capital letters (A–C) within each column represent significant differences between the means of the four types of cultivars. Different small letters (a–l, n) within each column represent significant differences between the means of each level of nitrogen input and cultivar. In both cases, the Tukey’s HSD test was used at *p* = 0.05.

**Table 4:** Retention time (Rt), wavelengths of maximum absorption in the visible region (*λ*_max_), mass spectral data and tentative identification of the phenolic compounds present in the hydroethanolic extracts of basil leaves in relation to nitrogen input.

Peak	Rt (min)	λ_max_ (nm)	[M-H]^−^ (*m*/*z*)	MS^2^ (*m*/*z*)	Tentative Identification
**1**	8.91	323	179	135(100)	Caffeic acid
**2**	14.96	323	473	313(61), 293(100)	Chicoric acid
**3**	16.8	334	609	301(100)	Quercetin-*O*-deoxyhexoside-hexoside
**4**	19.5	290/325	535	491(100), 287(34)	Eriodictyol-*O*-malonylhexoside
**5**	20.76	282/327	719	359(100), 197(31), 179(42), 161(50), 135(5)	Sagerinic acid
**6**	35.36	287/333	313	269(51), 203(12), 179(5), 161(100), 135(5)	Salvianolic acid F

**Table 5:** Quantification (mg/g of extract) of the phenolic compounds present in the hydroethanolic extracts of the studied basil genotypes in relation to nitrogen input (mean ± SD, *n* = 3).

Cultivar	ppm	Peak						TPA	TF	TPC
		1	2	3	4	5	6			
Dark Opal	0	1.6 ± 0.6 ^A,^*	**2 ± 1 ^A^**	**1 ± 2 ^A,B^**	**6 ± 2 ^A^**	**11 ± 8 ^A^**	**2.9 ± 0.5 ^A^**	**18 ± 10 ^A^**	**7 ± 4 ^A^**	**25 ± 13 ^A^**
		2.55 ± 0.07 ^b^	2.84 ± 0.01 ^c^	3.58 ± 0.05 ^a^	8.0 ± 0.4 ^c^	24.6 ± 0.4 ^a^	3.49 ± 0.06 ^c^	33.5 ± 0.2 ^b^	11.6 ± 0.5 ^b^	45.0 ± 0.3 ^b^
	200	1.51 ± 0.06 ^e^	3.98 ± 0.06 ^a^	0.275 ± 0.006 ^i^	8.1 ± 0.1 ^c^	10.56 ± 0.03 ^d^	3.17 ± 0.02 ^d^	19.2 ± 0.1 ^d^	8.4 ± 0.1 ^c^	27.6 ± 0.2 ^d^
	400	1.59 ± 0.01 ^d^	0.761 ± 0.003 ^h^	tr	3.86 ± 0.06 ^f^	5.08 ± 0.09 ^f^	2.33 ± 0.01 ^g^	9.76 ± 0.08 ^f^	3.86 ± 0.06 ^g^	13.62 ± 0.02 ^g^
	600	0.901 ± 0.007 ^j^	1.73 ± 0.04 ^e^	tr	3.1 ± 0.2 ^g^	4.61 ± 0.03 ^h,i^	2.43 ± 0.03 ^f^	9.67 ± 0.05 ^f^	3.1 ± 0.2 ^h^	12.8 ± 0.1 ^h^
Red Basil	0	**1.7 ± 0.8 ^A^**	**2 ± 1 ^A^**	**1 ± 1 ^A^**	**7 ± 4 ^A^**	**11 ± 8**	**3 ± 2 ^A^**	**18 ± 12 ^A^**	**8 ± 5 ^A^**	**25 ± 17 ^A^**
		2.68 ± 0.04 ^a^	3.06 ± 0.02 ^b^	3.55 ± 0.01 ^b^	11.5 ± 0.4 ^a^	23.2 ± 0.4 ^b^	4.93 ± 0.04 ^b^	33.9 ± 0.4 ^a^	15.1 ± 0.4 ^a^	48.9 ± 0.8 ^a^
	200	2.23 ± 0.02 ^c^	2.12 ± 0.02 ^d^	0.226 ± 0.001 ^j^	8.4 ± 0.3 ^b^	14.9 ± 0.4 ^c^	4.93 ± 0.03 ^b^	24.2 ± 0.4 ^c^	8.6 ± 0.3 ^c^	32.8 ± 0.7 ^c^
	400	1.15 ± 0.01 ^h^	1.00 ± 0.01 ^g^	0.34 ± 0.01 ^g^	4.43 ± 0.02 ^e^	4.43 ± 0.02 ^g^	1.227 ± 0.005 ^l^	7.807 ± 0.004 ^h^	4.77 ± 0.03 ^f^	12.58 ± 0.03 ^h^
	600	0.61 ± 0.02 ^n^	0.51 ± 0.01 ^j^	0.074 ± 0.004 ^k^	1.74 ± 0.09 ^i^	2.782 ± 0.03 ^l^	1.12 ± 0.03 ^m^	5.0 ± 0.1 ^j^	1.82 ± 0.09 ^j^	6.8 ± 0.1 ^l^
Basilico Rosso	0	**0.9 ± 0.2 ^B^**	**0.9 ± 0.2 ^B^**	**1 ± 1 ^A^**	**4 ± 1 ^B^**	**3.8 ± 0.7 ^B^**	**1.6 ± 0.4 ^B^**	**7 ± 1 ^B^**	**5 ± 2 ^B^**	**12 ± 3 ^B^**
		1.20 ± 0.02 ^g^	1.00 ± 0.01 ^g^	2.89 ± 0.01 ^c^	4.42 ± 0.07 ^e^	4.42 ± 0072 ^h^	1.46 ± 0.04 ^k^	8.4 ± 0.2 ^g^	7.3 ± 0.1 ^d^	15.7 ± 0.2 ^f^
	200	1.08 ± 0.01 ^i^	1.03 ± 0.02 ^f^	0.41 ± 0.01 ^f^	5.1 ± 0.2 ^d^	3.98 ± 0.05 ^j^	2.12 ± 0.02 ^h^	8.20 ± 0.01 ^g^	5.5 ± 0.2 ^e^	13.7 ± 0.2 ^g^
	400	0.726 ± 0.003 ^m^	1.01 ± 0.01 ^f,g^	0.624 ± 0.005 ^d^	3.2 ± 0.2 ^g^	3.7 ± 0.1 ^k^	1.88 ± 0.04 ^i^	7.3 ± 0.1 ^h^	3.8 ± 0.2 ^g^	11.1 ± 0.3 ^i^
	600	0.80 ± 0.03 ^l^	0.59 ± 0.02 ^i^	0.298 ± 0.004 ^h^	2.32 ± 0.01 ^h^	2.7 ± 0.1 ^l^	1.05 ± 0.01 ^n^	5.17 ± 0.03 ^j^	2.61 ± 0.01 ^i^	7.8 ± 0.1 ^k^
Mitikas	0	**1.0 ± 0.4 ^B^**	**0.8 ± 0.6 ^B^**	**-**	**1 ± 1 ^C^**	**3 ± 3 ^B^**	**3 ± 2 ^A^**	**8 ± 5 ^B^**	**2 ± 1 ^C^**	**9 ± 6 ^C^**
		1.34 ± 0.03 ^f^	1.72 ± 0.02 ^e^	0.52 ± 0.01 ^e^	3.22 ± 0.03 ^g^	7.6 ± 0.5 ^e^	4.97 ± 0.02 ^a^	15.6 ± 0.5 ^e^	3.74 ± 0.04 ^g^	19.4 ± 0.5 ^e^
	200	1.31 ± 0.01 ^f^	0.45 ± 0.01 ^k^	tr	1.49 ± 0.01 ^j^	2.21 ± 0.03 ^m^	3.02 ± 0.04 ^e^	6.99 ± 0.01 ^i^	1.49 ± 0.01 ^k^	8.48 ± 0.01 ^j^
	400	0.84 ± 0.02 ^k^	0.51 ± 0.01 ^j^	tr	1.08 ± 0.01 ^k^	1.77 ± 0.06 ^n^	1.50 ± 0.02 ^j^	4.6 ± 0.1 ^k^	1.08 ± 0.01 ^l^	5.7 ± 0.1 ^m^
	600	0.33 ± 0.02 ^o^	0.41 ± 0.01 ^l^	tr	0.065 ± 0.006 ^l^	0.98 ± 0.01 ^o^	1.25 ± 0.02 ^l^	3.0 ± 0.1 ^l^	0.065 ± 0.006 ^m^	3.0 ± 0.1 ^n^

tr—traces; TPA—total phenolic acids; TF—total flavonoids; TPC—tTotal phenolic compounds. * Different capital letters (A–C) within each column represent significant differences between the means of the four types of cultivars. Different small letters (a–o) within each column represent significant differences between the means of each level of nitrogen input and cultivar. In both cases the Tukey’s HSD test was used at *p* = 0.05. Calibration curves used: 1 and 2—caffeic acid (*y* = 388345*x* + 406369, *R*^2^ = 0.9939); 3 and 4—quercetin-3-*O*-rutinoside (*y* = 13343*x* + 76751, *R*^2^ = 0.9998); 5 and 6—rosmarinic acid (*y* = 191291*x* − 652903, *R*^2^ = 0.999).

**Table 6:** Antioxidant activity of the leaves’ hydroethanolic extracts of the studied basil genotypes in relation to nitrogen input (mean ± SD, *n* = 3).

Cultivar	ppm	TBARS (EC_50_, µg/mL)	OxHLIA (IC_50_ Values, µg/mL)	
			Δ*t* = 60 min	Δ*t* = 120 min
Dark Opal	0	32 ± 16 ^D,^*	**106 ± 56 ^B^**	**198 ± 89 ^B^**
		34 ± 3 ^d^	79 ± 3 ^g^	145 ± 4 ^f^
	200	13.1 ± 0.3 ^g^	30.8 ± 0.9 ^l^	82 ± 1 ^i^
	400	25.7 ± 0.5 ^f^	144 ± 2 ^d^	270 ± 3 ^d^
	600	55.4 ± 0.6 ^b^	171 ± 4 ^c^	293 ± 4 ^c^
Red Basil	0	**50 ± 15 ^B^**	**68 ± 26 ^D^**	**170 ± 84 ^C^**
		55.7 ± 0.2 ^b^	60 ± 2 ^h^	109 ± 2 ^h^
	200	25.2 ± 0.2 ^f^	40.0 ± 0.9 ^j,k^	79 ± 3 ^i^
	400	60 ± 1 ^a^	64 ± 3 ^h^	202 ± 4 ^e^
	600	61 ± 2 ^a^	109 ± 6 ^f^	289 ± 6 ^c^
Basilico Rosso	0	**43 ± 12 ^C^**	**77 ± 44 ^C^**	**151 ± 76 ^D^**
		32 ± 1 ^d^	38 ± 1 ^k,l^	65 ± 2 ^j^
	200	31.0 ± 0.2 ^e^	46 ± 3 ^i,j^	136 ± 3 ^f,g^
	400	54.9 ± 0.7 ^b^	147 ± 4 ^d^	271 ± 7 ^d^
	600	55.1 ± 0.8 ^b^	79 ± 3 ^g^	130 ± 5 ^g^
Mitikas	0	**57 ± 4 ^A^**	**183 ± 106 ^A^**	**269 ± 254 ^A^**
		54.3 ± 0.8 ^b^	51 ± 2 ^i^	105 ± 2 ^h^
	200	52.0 ± 0.9 ^c^	119 ± 7 ^e^	314 ± 15 ^b^
	400	59.7 ± 0.5 ^a^	250 ± 11 ^b^	na
	600	61.2 ± 0.2 ^a^	313 ± 13 ^a^	655 ± 19 ^a^
Trolox		5.4 ± 0.3	19.6 ± 0.7	41 ± 1

* Different capital letters (A–D) within each column represent significant differences between the means of the four types of cultivars. Different small letters (a–l) within each column represent significant differences between the means of each level of nitrogen input and cultivar. In both cases the Tukey’s HSD test was used at *p* = 0.05. na: not available.

**Table 7:** Antibacterial activity (minimal inhibition concentration (MIC) and minimal bactericidal concentration (MBC) mg/mL) of the leaves’ hydroethanolic extracts of the studied basil genotypes in relation to nitrogen input.

Cultivar	Nitrogen Level (ppm)	MIC/MBC	*S. aureus*	*B. cereus*	*L. monocytogenes*	*E. coli*	*S. Typhimurium*	*E. cloacae*
Dark Opal	0	**MIC**	4	1	2	2	2	4
		**MBC**	8	2	4	4	4	8
	200	**MIC**	2	1	1	2	2	2
		**MBC**	4	2	2	4	4	4
	400	**MIC**	1	1	2	1	2	1
		**MBC**	2	2	4	2	4	2
	600	**MIC**	2	1	2	2	2	2
		**MBC**	4	2	4	4	4	4
Red Basil	0	**MIC**	2	1	2	1	1	2
		**MBC**	4	2	4	2	2	4
	200	**MIC**	1	1	1	1	2	1
		**MBC**	2	2	2	2	4	2
	400	**MIC**	2	1	2	2	1	2
		**MBC**	4	2	4	4	2	4
	600	**MIC**	2	0.5	2	1	1	2
		**MBC**	4	1	4	2	2	4
Basilico Rosso	0	**MIC**	1	1	1	2	2	1
		**MBC**	2	2	2	4	4	2
	200	**MIC**	1	0.5	1	2	1	1
		**MBC**	2	1	2	4	2	2
	400	**MIC**	2	1	2	2	2	2
		**MBC**	4	2	2	4	4	4
	600	**MIC**	1	1	2	2	2	1
		**MBC**	2	2	4	4	4	2
Mitikas	0	**MIC**	2	1	2	2	2	2
		**MBC**	4	2	4	4	4	4
	200	**MIC**	2	1	2	2	1	2
		**MBC**	4	2	4	4	2	4
	400	**MIC**	2	1	2	2	1	2
		**MBC**	4	2	4	4	2	4
	600	**MIC**	2	1	2	1	2	2
		**MBC**	4	2	4	2	4	4
**Positive controls**	**E211**	**MIC**	4.0	0.5	1.0	1.0	1.0	2.0
		**MBC**	4.0	0.5	2.0	2.0	2.0	4.0
	**E224**	**MIC**	1.0	2.0	0.5	0.5	1.0	0.5
		**MBC**	1.0	4.0	1.0	1.0	1.0	0.5

**Table 8:** Antifungal activity (MIC and minimal fungicidal concentration (MFC) mg/mL) of the leaves’ hydroethanolic extracts (mg/mL) of the studied basil genotypes in relation to nitrogen input.

Cultivar	Nitrogen Level (ppm)	MIC/MFC	*A. fumigatus*	*A. niger*	*A. versicolor*	*P. funiculosum*	*P. v. var. cyclopium*	*T. viride*
Dark Opal	0	**MIC**	0.5	0.5	0.5	0.5	0.5	0.25
		**MFC**	1	1	1	1	1	0.5
	200	**MIC**	0.5	0.5	0.5	0.5	0.5	0.25
		**MFC**	1	1	1	1	1	0.5
	400	**MIC**	0.5	0.5	0.5	0.5	0.5	0.25
		**MFC**	1	1	1	1	1	0.5
	600	**MIC**	0.25	0.5	0.5	0.5	0.5	0.25
		**MFC**	0.5	1	1	1	1	0.5
Red Basil	0	**MIC**	0.5	0.5	0.5	0.5	0.5	0.25
		**MFC**	1	1	1	1	1	0.5
	200	**MIC**	0.5	0.5	0.5	0.5	0.5	0.25
		**MFC**	1	1	1	1	1	0.5
	400	**MIC**	0.25	0.5	0.25	0.5	1	0.25
		**MFC**	0.5	1	0.5	1	2	0.5
	600	**MIC**	0.5	0.5	0.5	0.5	0.5	0.25
		**MFC**	1	1	1	1	1	0.5
Basilico Rosso	0	**MIC**	0.25	0.5	0.5	0.5	0.5	0.25
		**MFC**	0.5	1	1	1	1	0.5
	200	**MIC**	0.5	0.5	0.5	1	1	0.5
		**MFC**	1	1	1	2	2	1
	400	**MIC**	0.25	0.5	0.25	0.5	1	0.25
		**MFC**	0.5	1	0.5	1	2	0.5
	600	**MIC**	0.5	0.5	0.5	0.5	0.5	0.25
		**MFC**	1	1	1	1	1	0.5
Mitikas	0	**MIC**	0.5	0.5	0.5	0.5	0.5	0.125
		**MFC**	1	1	1	1	1	0.25
	200	**MIC**	0.5	0.5	0.5	0.5	0.5	0.25
		**MFC**	1	1	1	1	1	0.5
	400	**MIC**	0.5	0.5	0.5	0.5	0.5	0.25
		**MFC**	1	1	1	1	1	0.5
	600	**MIC**	0.25	0.5	0.5	0.5	1	0.25
		**MFC**	0.5	1	1	1	2	0.5
**Positive** **controls**	**E211**	**MIC**	1.0	1.0	2.0	1.0	2.0	1.0
		**MFC**	2.0	2.0	2.0	2.0	4.0	2.0
	**E224**	**MIC**	1.0	1.0	1.0	0.5	1.0	0.5
		**MFC**	1.0	1.0	1.0	0.5	1.0	0.5

## Supplementary Materials

The following are available online at https://www.mdpi.com/2076-3921/9/11/1036/s1, Table S1: Parameter estimates, their significance, and adjusted R2 for the function representing the leaf, stem, and above-ground plant fresh weight to nitrogen input relationships in Dark Opal, Red Basil, Basilico Rosso, and Mitikas; Table S2: Fatty acids composition (%) of the studied basil genotypes in relation to nitrogen level (0–600 ppm) (mean ± SD, *n* = 3).
