# Supplementary material for: The Effect of Nitrogen Input on Chemical Profile and Bioactive Properties of Green- and Red-Colored Basil Cultivars
Source: Antioxidants (Basel). 2020 Oct 23;9(11):1036. doi: 10.3390/antiox9111036 (PMC7690662; doi:10.3390/antiox9111036)
Supplement: Supplementary file 1 [file antioxidants-09-01036-s001.pdf]

Supplementary material

Table S1. Parameter estimates, their significance, and adjusted R^2 for the function representing the leaf, stem, and above-ground plant fresh weight to nitrogen input relationships in Dark Opal, Red Basil, Basilico Rosso, and Mitikas.

**Cultivar**	**Fresh yield (g/plant)**	**Intercept**		**Slope**		**Quadratic coefficient**		**Adj R^2**
		**Estimate**	***p*-value**	**Estimate**	***p*-value**	**Estimate**	***p*-value**	
Dark Opal	Leaves	18.05	*0.0001*	0.0539	*0.0001*	-0.000079	*0.0001*	*0.81*
	Stem	5.02	*0.0001*	0.0069	*0.34*	-0.000013	*0.25*	*-0.01*
	Total	23.07	*0.0001*	0.0607	*0.0001*	-0.000092	*0.0001*	*0.50*
Red Basil	Leaves	21.70	*0.0001*	0.0329	*0.0001*	-0.000031	*0.006*	*0.69*
	Stem	5.10	*0.0001*	0.0204	*0.003*	-0.000030	*0.009*	*0.21*
	Total	26.80	*0.0001*	0.0533	*0.0001*	-0.000061	*0.001*	*0.59*
Basilico Rosso	Leaves	14.43	*0.0001*	0.0647	*0.0001*	-0.000098	*0.0001*	*0.86*
	Stem	5.05	*0.0001*	0.0047	*0.27*	-0.000011	*0.10*	*0.12*
	Total	19.48	*0.0001*	0.0694	*0.0001*	-0.000109	*0.0001*	*0.74*
Mitikas	Leaves	34.41	*0.0001*	0.1586	*0.0001*	-0.000227	*0.0001*	*0.76*
	Stem	4.66	*0.0001*	0.0301	*0.0001*	-0.000047	*0.0001*	*0.63*
	Total	39.07	*0.0001*	0.1886	*0.0001*	-0.000274	*0.0001*	*0.79*

Table S2. Fatty acids composition (%) of the studied basil genotypes in relation to nitrogen level (0–600 ppm) (mean ± SD, *n* = 3).

	**Dark Opal**				**Red Basil**			
	**0**	**200**	**400**	**600**	**0**	**200**	**400**	**600**
C12:0	0.052±0.005	0.068±0.001	0.096±0.003	0.114±0.001	0.194±0.007	0.125±0.001	0.145±0.004	0.144±0.003
C13:0	1.14±0.02	3.48±0.04	2.53±0.01	2.33±0.01	1.04±0.07	2.70±0.04	2.44±0.01	2.47±0.06
C14:0	0.242±0.007	0.358±0.008	0.240±0.005	0.249±0.003	0.353±0.001	0.332±0.001	0.239±0.004	0.249±0.006
C15:0	0.130±0.007	0.297±0.006	0.347±0.004	0.370±0.006	0.244±0.003	0.280±0.006	0.370±0.007	0.344±0.003
C15:1	0.39±0.01	0.273±0.009	0.86±0.03	0.869±0.008	0.48±0.01	0.877±0.002	0.647±0.001	0.624±0.003
C16:0	20.9±0.2	21.8±0.2	20.5±0.7	19.87±0.07	18.89±0.05	19.4±0.1	18.06±0.01	17.78±0.08
C16:1	2.15±0.01	4.17±0.08	2.76±0.02	2.7±0.1	3.58±0.08	2.46±0.01	3.28±0.06	3.44±0.02
C17:0	1.49±0.01	1.56±0.01	1.54±0.02	1.67±0.03	3.8±0.2	1.61±0.04	2.43±0.04	2.04±0.15
C18:0	7.5±0.1	4.09±0.05	4.1±0.2	3.8±0.3	3.26±0.02	3.73±0.01	3.38±0.07	3.16±0.04
C18:1n9c	4.3±0.1	3.02±0.09	3.48±0.07	3.46±0.04	3.37±0.02	3.30±0.06	3.09±0.01	3.72±0.01
C18:2n6c	14.6±0.4	15.2±0.2	13.4±0.4	14.4±0.4	14.17±0.07	14.06±0.07	14.29±0.06	15.13±0.01
C18:3n3	44.6±0.3	41.8±0.1	48.9±0.2	49.08±0.04	48.6±0.4	50.03±0.06	50.08±0.07	49.3±0.1
C20:2	0.56±0.04	0.82±0.05	0.55±0.01	0.53±0.01	0.606±0.004	0.445±0.003	0.52±0.05	0.55±0.03
C23:0	0.63±0.05	2.37±0.01	0.39±0.04	0.373±0.009	0.759±0.008	0.373±0.008	0.63±0.02	0.537±0.008
C24:0	1.27±0.03	0.729±0.001	0.37±0.01	0.315±0.001	0.639±0.007	0.344±0.001	0.41±0.02	0.524±0.001
SFA	32.1±0.1	34.1±0.2	29.7±0.6	28.8±0.4	28.5±0.2	28.5±0.1	27.69±0.02	26.7±0.1
MUFA	8.2±0.1	8.2±0.2	7.5±0.1	7.3±0.1	8.12±0.1	7.0±0.1	7.43±0.04	8.31±0.02
PUFA	59.7±0.2	57.8±0.4	62.8±0.6	64.0±0.5	63.3±0.4	64.5±0.1	64.9±0.1	65.0±0.1

Table S2
Cont.

	Basilico Rosso				Mitikas			
	**0**	**200**	**400**	**600**	**0**	**200**	**400**	**600**
C12:0	0.174±0.001	0.159±0.006	0.198±0.006	0.162±0.004	0.195±0.003	0.160±0.006	0.161±0.002	0.179±0.001
C13:0	0.539±0.006	2.35±0.01	2.94±0.01	3.26±0.09	2.27±0.07	2.76±0.01	2.77±0.05	3.03±0.01
C14:0	0.669±0.007	0.37±0.01	0.344±0.002	0.365±0.004	0.500±0.008	0.260±0.008	0.29±0.02	0.337±0.006
C15:0	0.248±0.008	0.251±0.001	0.240±0.009	0.39±0.01	0.789±0.009	0.370±0.008	0.356±0.002	0.315±0.002
C15:1	0.465±0.001	0.835±0.001	0.541±0.009	1.07±0.03	0.62±0.03	0.95±0.01	1.05±0.01	0.87±0.05
C16:0	21.5±0.5	18.43±0.01	20.5±0.8	20.3±0.6	27.36±0.01	19.12±0.06	19.8±0.2	21.2±0.6
C16:1	3.939±0.009	2.67±0.04	3.52±0.01	3.8±0.1	10.5±0.1	7.14±0.01	7.05±0.07	6.66±0.23
C17:0	1.04±0.01	1.44±0.02	0.742±0.001	0.648±0.007	0.506±0.004	0.363±0.001	0.340±0.005	0.343±0.001
C18:0	5.14±0.01	3.86±0.02	3.84±0.02	3.76±0.06	6.16±0.03	2.70±0.08	2.97±0.01	3.045±0.003
C18:1n9c	4.18±0.08	3.64±0.02	4.15±0.01	3.15±0.02	4.0±0.2	2.18±0.07	2.58±0.07	2.63±0.05
C18:2n6c	15.5±0.2	13.3±0.1	15.2±0.1	13.17±0.05	13.4±0.3	14.0±0.6	14.45±0.04	13.7±0.2
C18:3n3	45.4±0.4	51.0±0.2	45.5±0.7	47.3±0.5	31.3±0.1	48.5±0.5	46.5±0.1	45.7±0.6
C20:2	0.311±0.004	0.85±0.03	1.00±0.01	1.27±0.05	0.68±0.04	0.434±0.002	0.466±0.002	0.630±0.008
C23:0	0.737±0.007	0.37±0.02	1.00±0.02	0.97±0.01	0.710±0.007	0.500±0.006	0.62±0.03	0.62±0.03
C24:0	0.259±0.004	0.450±0.005	0.299±0.004	0.39±0.03	1.00±0.01	0.529±0.008	0.61±0.04	0.67±0.01
SFA	30.0±0.5	27.2±0.1	29.8±0.8	29.9±0.6	38.5±0.1	26.2±0.1	27.3±0.2	29.1±0.6
MUFA	8.8±0.1	7.6±0.1	8.5±0.1	8.4±0.1	16.2±0.3	10.8±0.1	11.3±0.1	10.8±0.2
PUFA	61.2±0.6	65.2±0.1	61.7±0.8	61.8±0.6	45.3±0.3	63.0±0.1	61.4±0.1	60.1±0.8
